# The dual IGF-1R/IR blocker, Linsitinib, modulates human orbital fibroblast activity and proliferation

**DOI:** 10.3389/fimmu.2026.1920590

**Published:** 2026-10-02

**Authors:** Maximilian Luffy, Stefanie Wagner, Jan Wolf, Anna-Lena Ganz, Xaver Michels, Ryan Zeidan, Jeffrey D. Kent, Raymond S. Douglas, George J. Kahaly

**Affiliations:** 1 Molecular Thyroid Research Laboratory, Department of Medicine I, Johannes Gutenberg-University (JGU) Medical Center, Mainz, Germany; 2 Sling Therapeutics, Ann Arbor, MI, United States

**Keywords:** apoptosis, IGF-1 receptor, insulin receptor, Linsitinib, orbital fibroblast, thyroid eye disease, tyrosine kinase inhibitor

## Abstract

**Aim:**

The insulin-like growth factor-1 receptor (IGF-1R) and its downstream signaling pathways drive proliferation, survival, and extracellular matrix production, representing key therapeutic targets in thyroid eye disease (TED). This novel *in vitro* study investigated the inhibitory effects of the dual IGF-1R/insulin receptor small-molecule inhibitor Linsitinib on orbital target cell activity, proliferation, and survival.

**Methods:**

Human orbital fibroblasts (OF) were pre-treated with increasing doses of Linsitinib and stimulated with 100 ng/mL IGF-1 to quantify hyaluronic acid (HA) and pro-collagen in supernatants via ELISA. Intracellular protein kinase B (AKT) phosphorylation at Serine 473 was quantified in cell lysates. Additionally, an equimolar comparison between Linsitinib and the anti-IGF-1R monoclonal antibody Teprotumumab was performed in IGF-1R-expressing cells. Proliferation was assessed both microscopically and by tetrazolium assay, while apoptosis was evaluated by caspase-3/7 activity.

**Results:**

Linsitinib markedly suppressed IGF-1-induced HA and pro-collagen secretion, with TED OF showing significantly greater sensitivity and exclusive morphological evidence of apoptosis compared to control OF. Linsitinib effectively inhibited IGF-1-induced AKT phosphorylation to baseline levels in OF. Equimolar comparison in IGF-1R-expressing cells demonstrated that Linsitinib outperformed Teprotumumab in inhibiting cell proliferation across most tested concentrations, whereas Teprotumumab showed higher potency only at the lowest concentrations. While Teprotumumab failed to induce cell death, Linsitinib triggered robust activity of the apoptosis markers caspase-3/7. Morphologically, in contrast to Teprotumumab, Linsitinib induced dose-dependent changes characterized by cellular contraction and formation of gaps in the monolayer.

**Conclusion:**

Linsitinib reverses OF activation through intracellular kinase blockade while outperforming Teprotumumab in equimolar comparison. These findings highlight the role of Linsitinib as a potent, dual-targeted therapy for TED.

## 1 Introduction

The ubiquitously expressed insulin-like growth factor-1 receptor (IGF-1R) and its primary ligand IGF-1 are essential for regulating the proliferation, survival, and metabolic functions of numerous cell types (1, 2). This receptor kinase has a heterotetrameric structure composed of two identical extracellular α-subunits and two identical transmembrane β-subunits connected by disulfide bonds (3–5). Each α-subunit contains a cysteine-rich domain that serves as the primary ligand-binding site (5–7). The β-subunits consist of a short extracellular domain, a 24-residue hydrophobic transmembrane domain, and a larger cytoplasmic region containing a kinase domain that shares 84% amino acid sequence homology with the kinase domain of the insulin receptor (IR) (8, 9). The intracellular region of the β-subunit contains an ATP-binding site at lysine 1003 (7). The kinase domain encompasses the activation loop made up of three critical tyrosine residues (Tyr1131, Tyr1135, and Tyr1136), which is phosphorylated upon ligand binding (1). Once activated, phosphorylated tyrosine 950 provides a docking site for substrates such as IR substrates 1–4 (IRS-1–4) and Src homology and collagen (SHC) proteins (9). These substrates trigger downstream signaling via the phosphoinositide 3-kinase/protein kinase B (PI3K/AKT) pathway, which mediates cell growth, cell migration, and cell survival (10, 11), as well as the mitogen-activated protein kinase/extracellular signal-regulated kinase (MAPK/ERK) pathway, which regulates cell proliferation, differentiation, cell cycle, and cell survival (12, 13).

The IGF-1R, co-localized with the thyrotropin receptor (TSH-R) in orbital fibroblasts (OF), plays a critical role in the pathogenesis of thyroid eye disease (TED), the most common extra-thyroidal manifestation of Graves’ disease (GD) (14), and represents an effective therapeutic target (4, 15–18). Several anti-IGF-1R blockers, including monoclonal antibodies (mAbs) and small-molecule inhibitors, have been evaluated in pre-clinical studies and human clinical trials (19–21). These two classes of inhibitors have distinct characteristics and pharmacokinetic properties.

Small-molecule drugs are synthetic chemicals with a low molecular weight (typically <700 Da) and are administered orally (22). MAbs are large proteins (approximately 150 kDa), requiring intravenous and/or subcutaneous administration (22, 23). The larger size of mAbs limits their utility to extracellular targets, while small molecules can translocate through plasma membranes to interact with the cytoplasmic domain of cell-surface receptors and intracellular signaling molecules (22). Compared to the relatively short half-life of small molecules (4–24 h), mAbs have a longer half‐life (11–30 days) in humans (23). Treatment of TED patients with Teprotumumab, a fully human mAb targeting the IGF-1R, markedly reduced proptosis while improving diplopia and quality of life (24, 25).

Linsitinib is a potent, selective small molecule that acts as a dual ATP-competitive kinase inhibitor of both IGF-1R and IR (26, 27). It binds directly to the intracellular ATP-binding site of the kinase domain in an ATP-competitive fashion (28).

In this novel experimental study, the inhibitory effects of Linsitinib on hyaluronic acid (HA) and human pro-collagen secretion were evaluated in conditioned supernatants of human OF following stimulation with IGF-1. Furthermore, the impact of Linsitinib on cell proliferation and its ability to induce apoptosis were investigated in an IGF-1R-expressing cell line. To characterize the inhibitory potency of Linsitinib, an equimolar (1:1) comparison with Teprotumumab was performed in both assays to evaluate receptor-blocking efficiency on a molecule-by-molecule basis.

## 2 Materials and methods

### 2.1 Cell culture

#### 2.1.1 Primary human orbital fibroblasts

Primary human OF were established on a regular basis from collected fresh orbital tissue following written consent from patients with TED or control subjects devoid of autoimmune disease undergoing orbital decompression surgery at the JGU Medical Center. OF were maintained in 75 cm^2^ flasks containing 10 mL of medium at 37 °C and 5% CO_2_. The growth medium consisted of DMEM/Ham’s F-12 and DMEM high glucose (GlutaMax; both from Life Technologies, Dreieich, Germany), supplemented with 10% fetal bovine serum (PAN-Biotech, Aidenbach, Germany). Additional supplements included penicillin-streptomycin, fungizone, and gentamicin (all from Life Technologies); Y-27632 dihydrochloride (Tocris Bioscience, Bristol, UK); hydrocortisone, insulin, and cholera toxin (all from Sigma-Aldrich, St. Louis, MO, USA); and epidermal growth factor (EGF; PeproTech, Cranbury, NJ, USA). The medium was changed weekly, and all experiments were performed using cells at low passage numbers (≤ passage 3). For the described experiments, samples derived from patients with TED (n = 2) and controls (n = 2) were selected.

#### 2.1.2 IGF-1R cell line

The IGF-1R-expressing adrenocortical cell line NCI-H295R (ATCC, Manassas, VA, USA) was cultured in DMEM: F12 medium (ATCC) supplemented with 2.5% Nu-Serum (Corning, Corning, NY, USA) and ITS+ Premix (Corning) containing insulin, transferrin, selenium, bovine serum albumin, and linoleic acid. The medium was changed three times per week. As the IGF-1R cell line grows both as an adherent monolayer and in suspension, a specific retrieval protocol was followed according to the manufacturer’s instructions during medium changes to maintain total cell population. The medium from the culture flask was centrifuged at 700 rpm for 5 min to obtain a cell pellet, allowing the retrieval of cells in suspension, while fresh growth medium was added to the adherent cells. The resuspended pellet was then returned to the culture flask. Cultivation was continued in an incubator at 37 °C with 5% CO_2_.

#### 2.1.3 Ethics and consent to participate statement

All procedures in the current study involving human orbital tissue were performed in accordance with the Declaration of Helsinki. An agreement between the local Institutional Review Board and the JGU Medical Center allows the collection of remnant tissue for scientific purposes. Written informed consent was obtained from all patients.

### 2.2 Quantification of AKT phosphorylation in cell lysates of human orbital fibroblasts

#### 2.2.1 Preparation of orbital fibroblasts and cell lysates

Primary OF were detached from the culture flask at ≥90% confluence using Accutase for 15 min at 37 °C and 5% CO_2_. The cell suspension was centrifuged for 5 min at 2,000 rpm, and the obtained cell pellet was resuspended in starvation medium consisting of DMEM high glucose supplemented with 0.5% FBS and 1% penicillin-streptomycin solution. Viable cell counts were determined via an automated cell counter. OF were seeded in a 48-well plate at a density of 40,000 cells per well in 250 μL starvation medium and cultured for 24 h at 37 °C and 5% CO_2_. The following day, the cells were washed three times with PBS and pre-treated for 1 h with 250 μL of three (n = 3) rising concentrations of Linsitinib diluted in starvation medium. All conditions were performed in duplicate. For the stimulation phase, media were removed from all wells. Cells pre-treated with Linsitinib, as well as the cells in the positive control wells, were stimulated with 250 μL IGF-1 (100 ng/mL) in standard growth medium (DMEM/Ham’s F-12 and DMEM high glucose). The standard growth medium only served as the negative control. All conditions were incubated for 20 min (37 °C, 5% CO_2_) before continuing with the assay procedure (Section 2.2.2).

#### 2.2.2 AKT 1/2/3 (pS473) ELISA

AKT phosphorylation at Serine 473 (pS473) in cell lysates was quantified using a commercial sandwich ELISA (Abcam, Cambridge, UK) according to the manufacturer’s instructions. Following treatment in the 48-well plate, media were removed and cells were washed twice with PBS. For cell lysis, 150 μL of cell extraction buffer was added directly to each well and incubated for 20 min on ice.

Afterwards, lysed cells were scraped into test tubes and incubated on ice for an additional 15 min. After 20 min of centrifugation at 18,000×*g* (4 °C), lysate supernatants were transferred into clean test tubes. In the meantime, a seven-point standard series of AKT control lysate was prepared in extraction buffer. Briefly, 50 μL of each standard and sample were transferred to a pre-coated test plate, followed by the addition of 50 μL of the antibody (Ab) cocktail to each well. Standards and samples were processed in duplicate (n = 2).

The plate was incubated for 1 h at room temperature (RT) on a shaker at 400 rpm. Following three wash steps with 200 μL of wash buffer, 100 μL of tetramethylbenzidine (TMB) substrate was added and incubated for 15 min at RT protected from light. The enzymatic reaction was blocked by the addition of 100 μL of stop solution. Absorbance was immediately measured at 450 nm using a GloMax Discover Microplate Reader GM3000 (Promega, Madison, Wisconsin, USA). AKT (pS473) phosphorylation levels were interpolated from the control lysate standard curve.

### 2.3 Quantification of hyaluronic acid and pro-collagen in conditioned supernatants

#### 2.3.1 Preparation of orbital fibroblasts and supernatant collection

Primary OF were detached from the culture flask at ≥90% confluence using Accutase (Merck, Darmstadt, Germany) for 15 min at 37 °C and 5% CO_2_. The cell suspension was centrifuged for 5 min at 2,000 rpm, and the obtained cell pellet was resuspended in growth medium. Viable cell counts were determined using an automated cell counter (Logos Biosystems, Anyang-si, Gyeonggi-do, South Korea). OF were seeded in a 24-well plate at a density of 70,000 cells per well in 500 μL medium and cultured for 24 h at 37 °C and 5% CO_2_ to reach confluence. The following day, the cells were washed three times with PBS and pre-treated with 1 U/mL hyaluronidase (Sigma-Aldrich) in Hank’s Balanced Salt Solution (HBSS) for 1 h in the incubator to remove pre-existing HA. Following pre-treatment, the solution was decanted, and wells were washed twice with HBSS. For the inhibition phase, cells were incubated for 1 h with 500 μL of five (n = 5) rising concentrations of the IGF-1R blocker Linsitinib (Sling Therapeutics, Ann Arbor, MI, USA). IGF-1 (100 ng/mL; PeproTech) and medium only served as positive and negative controls, respectively. All conditions were performed in duplicate. For the stimulation phase, IGF-1 (100 ng/mL) was added to all wells pre-treated with Linsitinib and incubated for 48 h (37 °C, 5% CO_2_). Following incubation, treatment effects were microscopically documented as described in *Section 2.3.4*. To measure secreted HA and collagen during a defined period and ensure no interference from the compounds, the medium from all wells was replaced with fresh growth medium for a final 24 h incubation. The conditioned supernatants were collected and stored at −20 °C until analysis.

#### 2.3.2 Hyaluronic acid sandwich ELISA

HA concentrations in the conditioned supernatants were quantified using a commercial sandwich ELISA (Echelon Biosciences, Salt Lake City, UT, USA) according to the manufacturer’s instructions. The assay utilizes a capture Ab immobilized on a test plate to bind HA (antigen). After addition of a biotinylated detection Ab and streptavidin-conjugated horseradish peroxidase (HRP), a specific sandwich complex forms. The detection is based on the oxidation of TMB by HRP to a blue-colored product, quantified by absorbance. Supernatants and a five-point standard series were diluted 1:10 in assay buffer. Briefly, 100 μL of diluted standards were transferred to a pre-coated test plate. Standards were processed in duplicate (n = 2), and six replicates (n = 6) were performed for each sample. The test plate was incubated for 1 h at room temperature (RT) on a shaker at 150 rpm. After three wash steps with 200 μL of wash buffer, 100 μL of biotinylated HA-binding detection Ab was added to each well and incubated for 1 h at RT. Following three wash steps, 100 μL of TMB substrate was added and incubated for 10 min at RT protected from light. The enzymatic reaction was blocked by the addition of 50 μL of stop solution. Absorbance was immediately measured at 450 nm using a GloMax Discover Microplate Reader (GM3000; Promega, Madison, WI, USA). HA concentrations were interpolated from the standard curve of known HA concentrations.

#### 2.3.3 Pro-collagen sandwich ELISA

Pro-collagen concentrations in the conditioned supernatants were quantified using a commercial sandwich ELISA (Abcam, Cambridge, UK) according to the manufacturer’s instructions. The assay utilizes a capture Ab and an HRP-conjugated detection Ab that form a sandwich complex with pro-collagen (antigen). This complex is immobilized on the plate via an affinity tag on the capture Ab. Detection is based on the HRP-mediated oxidation of TMB to a blue-colored product, quantified by absorbance. A seven-point standard series was prepared in sample buffer. Supernatant samples were diluted 1:2,000 in sample buffer (0.5 μL sample + 999.5 μL sample). Briefly, 50 μL of each standard and sample were transferred to a pre-coated test plate, followed by the addition of 50 μL of the Ab cocktail to each well. Standards were processed in duplicate (n = 2), and six replicates (n = 6) were performed for each sample. The plate was incubated for 1 h at RT on a shaker at 250 rpm. Following three wash steps with 200 μL of wash buffer, 100 μL of TMB substrate was added and incubated for 10 min at RT protected from light. The enzymatic reaction was blocked by the addition of 100 μL of stop solution. Absorbance was immediately measured at 450 nm using GloMax Reader (Promega). Pro-collagen concentrations were interpolated from the standard curve of known pro-collagen concentrations.

#### 2.3.4 Morphological evaluation and imaging

Cell morphology and treatment effects were evaluated by phase-contrast microscopy using a ZEISS Primovert inverted microscope (Carl Zeiss, Oberkochen, Germany) equipped with a Plan-Achromat ×10/0.25 Ph1 objective and an Axiocam ERc 5s camera (Carl Zeiss). Spatial resolution data were captured with pixel dimensions of 2,560 pixels × 1,920 pixels and a lateral scaling (xy) of 0.220 μm/pixel. As images represent single time-point documentation, depth (z) and time (t) dimensions were not applicable. Images were acquired with a bit depth of 24 bits, a constant exposure time of 140.21 ms, and a binning factor of 1.1 using ZEISS ZEN lite software. The display lookup table (LUT) used for image presentation is linear and covers the full range of data.

### 2.4 Cell proliferation and apoptosis in primary orbital fibroblasts

#### 2.4.1 Proliferation assay

Cell proliferation was quantified in primary human OF using a colorimetric MTS assay (Promega). This assay utilizes a tetrazolium compound, which is reduced by metabolically active cells into a colored formazan product, proportional to the number of viable cells. The assay procedure extends over three days under aseptic conditions. Primary OF were detached from culture flasks at ≥80% confluence using 10 mL of Accutase (Merck, Darmstadt, Germany) for 10 min at 37 °C and 5% CO_2_. The cell suspension was centrifuged in a Falcon tube at 2,000 rpm for 5 min. Following resuspension, viable cells were determined by automated cell counting with acridine orange staining (Logos Biosystems). Cells were seeded at a density of 10,000 cells per well in 100 μL medium into the 48 inner wells of 96-well plates and cultivated for 24 h at 37 °C and 5% CO_2_ to reach confluence.

A ten-point dilution series of Linsitinib was initially prepared in dimethyl sulfoxide (DMSO) and subsequently diluted two times in growth medium to ensure a final DMSO concentration of <0.5% to avoid cytotoxicity. Afterwards, 100 μL of each concentration was transferred in eight replicates (n = 8) to the plates pre-filled with 100 μL growth medium, resulting in final concentrations ranging from 0.293 µM to 150 µM. For negative controls, 200 μL of medium only was added to the cells. For blank controls (medium without cells), 200 μL of medium was pipetted in duplicate. Following 24-h incubation with Linsitinib at 37 °C and 5% CO_2_, 40 μL of MTS reagent was added to each well and incubated for 4 h in the incubator. Absorbance was measured at 490 nm using a GloMax Reader (Promega).

#### 2.4.2 Caspase-Glo^®^ 3/7 assay (apoptosis)

Apoptosis was quantified in primary human OF using a Caspase-Glo 3/7 luminescence assay (Promega) to measure the activity of caspases 3 and 7 as markers for programmed cell death. The assay utilizes a proluminescent DEVD-aminoluciferin substrate, which is specifically cleaved by caspases 3 and 7. The resulting aminoluciferin is consumed by luciferase to generate a luminescent signal proportional to caspase activity. The experimental procedure followed the same protocol as the proliferation assay (Section 2.4.1) with the following modifications to volumes: On day 2, 50 μL of each Linsitinib concentration was transferred to plates pre-filled with 50 μL growth medium, resulting in a final volume of 100 μL per well. For negative controls, 100 μL of medium only was added to the cells. For blank controls (medium without cells) in duplicate, 100 μL of medium was pipetted. Following 24-h incubation with Linsitinib, 100 μL of Caspase-Glo 3/7 reagent was added to each well (1:1 ratio) and incubated for 30 min at RT protected from light. Luminescence was measured using a GloMax Reader (Promega).

### 2.5 Equimolar comparison of Linsitinib and Teprotumumab

#### 2.5.1 Proliferation assay

The equimolar comparison between Linsitinib and commercially available, synthesized Teprotumumab (BioVision, Milpitas, CA, USA) followed the same protocol as the proliferation assay (Section 2.4.1) with specific modifications: Experiments were performed using IGF-1R-expressing cells. At ≥80% confluence, cells were detached using 3 mL of trypsin (PAN-Biotech) for 10 min at 37 °C and 5% CO_2_. The resulting cell suspension was supplemented with growth medium and centrifuged at 700 rpm for 5 min. Cells were seeded and cultivated in 96-well plates for 24 h in an incubator.

To compare the inhibitory effects of Linsitinib and Teprotumumab, an equimolar (1:1) comparison was performed using an eight-point dilution series (37.5 µM to 0.293 µM). For Linsitinib, two additional concentrations (up to 150 µM) were included to expand analysis at high concentrations. Teprotumumab was directly diluted in growth medium.

Following 24-h incubation with the inhibitors at 37 °C and 5% CO_2_, cell morphology was documented microscopically as described in *Section 2.3.4*. Subsequently, cell proliferation was quantified by the MTS assay, and absorbance was measured using a GloMax Reader (Promega).

#### 2.5.2 Caspase-Glo^®^ 3/7 assay (apoptosis)

Apoptosis in IGF-1R-expressing cells was quantified via the Caspase-Glo 3/7 assay. Cell seeding and the equimolar dilution series of Linsitinib and Teprotumumab were executed as detailed in *Section 2.5.1*. To accommodate the Caspase-Glo 3/7 assay format, the volumes of the treatment solutions and the respective controls were modified to a final volume of 100 μL per well (50 μL of inhibitor solution with 50 μL of growth medium; 100 μL of medium for negative and blank controls), as outlined in Section 2.4.2.

### 2.6 Statistical analysis

Statistical analysis was performed using GraphPad Prism software version 10.4.0 (GraphPad Software, Boston, MA, USA). For all assays, statistical comparisons of each inhibitor concentration relative to control were calculated using the Kruskal–Wallis test followed by Dunn’s multiple comparisons test. Data of HA and pro-collagen assays are expressed as absolute concentrations (ng/mL and pg/mL, respectively) and presented as mean + SD. Data of cell proliferation and apoptosis assays are expressed relative to the untreated control and presented as mean + SD. For equimolar comparison, statistical significance between Linsitinib and Teprotumumab at each concentration within the proliferation assays was calculated using the two-tailed Mann–Whitney test. For all analyses, statistical significance was defined as P <0.05.

## 3 Results

### 3.1 Effect of Linsitinib on AKT phosphorylation in human OF

Linsitinib effectively inhibited the IGF-1-induced AKT phosphorylation at Serine 473 in primary OF from a TED patient in a clear dose-dependent manner. Stimulation with IGF-1 (100 ng/mL) induced a robust increase in AKT (pS473) phosphorylation levels relative to baseline. This stimulatory effect was inhibited by pre-treatment with Linsitinib (**Figure 1**). At the lowest tested concentration of 0.293 μM, Linsitinib reduced IGF-1-induced AKT phosphorylation by 21%. A moderate concentration of 9.38 μM suppressed AKT phosphorylation by 44%, while the highest concentration (150 μM) inhibited phosphorylation by 52%, returning AKT phosphorylation levels back to baseline.

**Figure 1:**
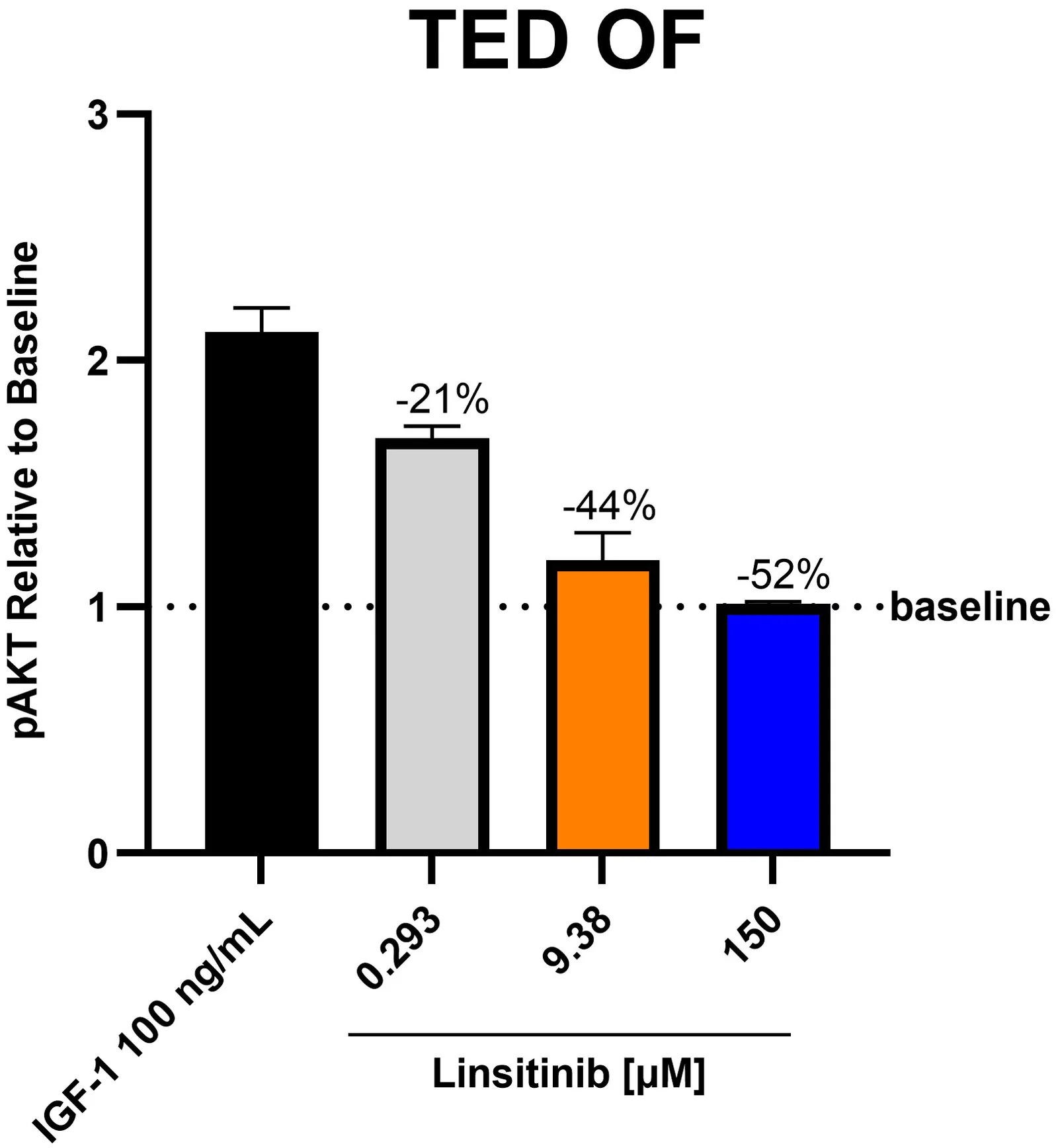
Linsitinib effectively inhibits IGF-1-induced AKT phosphorylation in human orbital fibroblasts. Semi-quantitative measurement of AKT phosphorylation in a cell lysate of primary human orbital fibroblasts (OF) from a TED patient. After pre-treatment with increasing concentrations of Linsitinib (0.293–150 μM) for 1 h, OF were stimulated with IGF-1 (100 ng/mL) for 20 min. Linsitinib suppressed IGF-1-induced AKT phosphorylation in a dose-dependent manner at 0.293 μM (−21%), 9.38 μM (−44%), and 150 μM (−52%), respectively. Data are expressed relative to the baseline level (untreated control) and presented as mean ± SD with n = 2 replicates per concentration. Percentage changes were calculated relative to the IGF-1 positive control.

### 3.2 Effect of Linsitinib on HA and pro-collagen release in human OF

#### 3.2.1 Linsitinib suppresses IGF-1-induced HA secretion

Linsitinib demonstrated potent inhibitory effects on IGF-1-induced HA release (**Figure 2**). While the stimulatory effects of IGF-1 were effectively reversed in OF derived from TED patients, the inhibitory response in control OF was less marked. Stimulation with 100 ng/mL IGF-1 led to a substantial increase in HA levels compared to baseline in a TED donor (**Figure 2A**). Linsitinib inhibited IGF-1-induced HA release up to 83% (P <0.0001). HA levels were effectively returned to baseline level at 9.38 μM (P = 0.0288). Higher Linsitinib concentrations further suppressed HA release to levels below baseline, at 75 μM (P = 0.0004) and 150 μM (P <0.0001).

**Figure 2:**
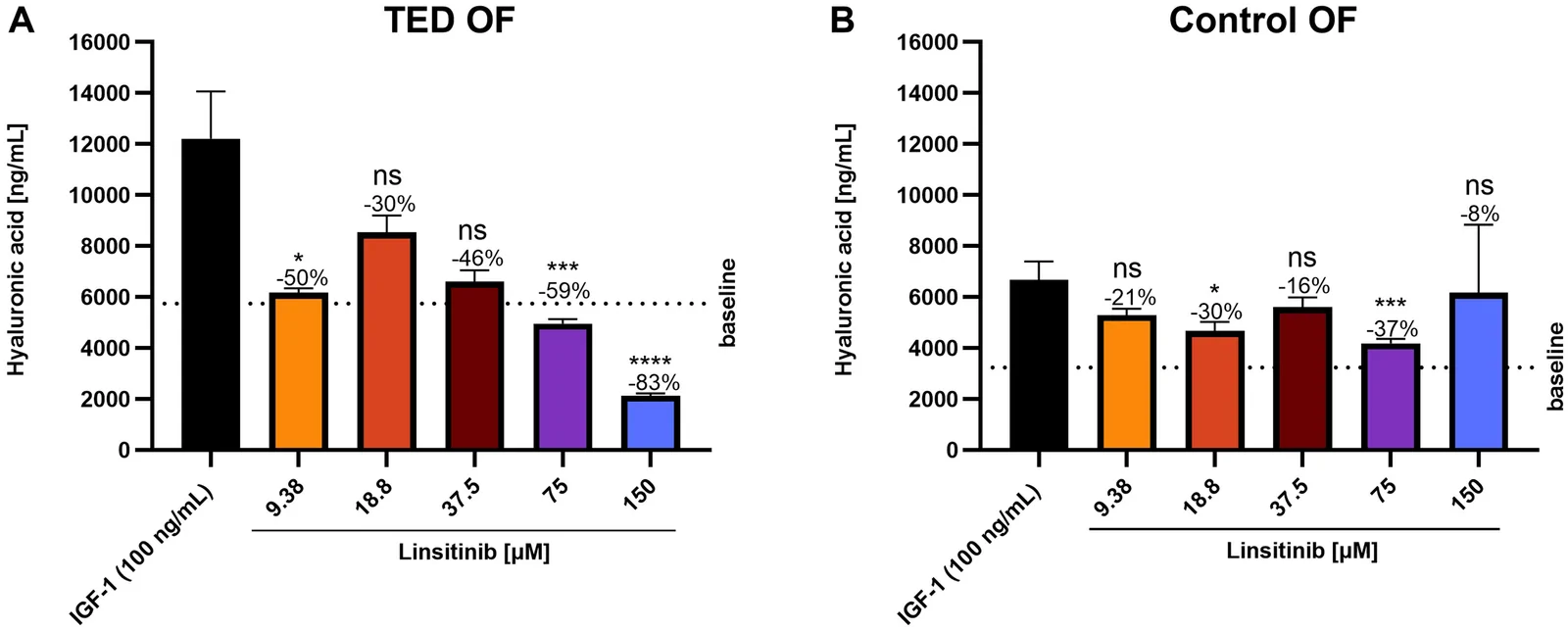
Linsitinib normalizes IGF-1-induced hyaluronan secretion with enhanced efficacy in TED orbital fibroblasts. Quantitative analysis of hyaluronic acid (HA) concentration in the supernatant of primary human orbital fibroblasts (OF) from a TED patient **(A)** and a control donor **(B)**. After incubation with increasing concentrations of Linsitinib (9.38–150 μM) for 1 h, OF were stimulated with IGF-1 (100 ng/mL) for 48 h. **(A)** In TED OF, Linsitinib returned HA levels to the baseline level at 9.38 μM (−50%, P = 0.0288). Higher concentrations of Linsitinib further suppressed HA release to levels below baseline, reaching inhibition of 59% at 75 μM (P = 0.0004) and 83% at 150 μM (P <0.0001). **(B)** In control OF, Linsitinib moderately suppressed HA release at 18.8 μM (−30%, P = 0.0128) and 75 μM (−37%, P = 0.0002). Data are expressed as absolute concentrations and presented as mean ± SD, with n = 6 replicates per concentration. Percentage changes and statistical significance were calculated relative to the IGF-1 positive control using the Kruskal–Wallis test followed by Dunn’s multiple comparisons test. ns, P>0.05. *P≤0.05. ***P≤0.001. ****P≤0.0001.

In comparison, the effect of Linsitinib was less marked in tested control donors, showing lower responses to IGF-1. The positive control induced only a minor increase in HA levels in a control donor (**Figure 2B**). Linsitinib treatment moderately suppressed this response, reaching a maximum inhibition of 37%. Linsitinib reduced HA release at 18.8 μM (P = 0.0128) and 75 μM (P = 0.0002).

In another TED donor, marked, robust stimulatory and inhibitory effects were observed (**Supplementary Figure 1A**). Linsitinib treatment suppressed this response, with inhibition up to 61% relative to the positive control. Specifically, Linsitinib restored HA secretion to baseline level at concentrations of 9.38 μM (P = 0.0027), 18.8 μM (P = 0.0330), 37.5 μM (P = 0.198), and 150 μM (P = 0.0022). The stimulatory effect of the positive control was more evident when testing another control donor (**Supplementary Figure 1B**). At lower concentrations, Linsitinib showed no inhibitory effect. Linsitinib reduced HA secretion below baseline at the highest concentration of 150 μM (P = 0.0197).

#### 3.2.2 Linsitinib reverses IGF-1-induced pro-collagen I release

Linsitinib demonstrated strong inhibitory effects on IGF-1-induced pro-collagen release (**Figure 3**). In contrast to the HA assay, Linsitinib markedly inhibited pro-collagen release across all donor groups. The stimulatory effects of IGF-1 were effectively reversed in both TED and control OF, with Linsitinib consistently restoring pro-collagen release to levels below baseline. A robust stimulatory effect of IGF-1 was observed in a TED donor (**Figure 3A**). Linsitinib treatment markedly suppressed this response up to 62%. Linsitinib reduced pro-collagen release below baseline in a dose-dependent trend at 37.5 μM (P = 0.0080), 75 μM (P = 0.0003), and 150 μM (P <0.0001).

**Figure 3:**
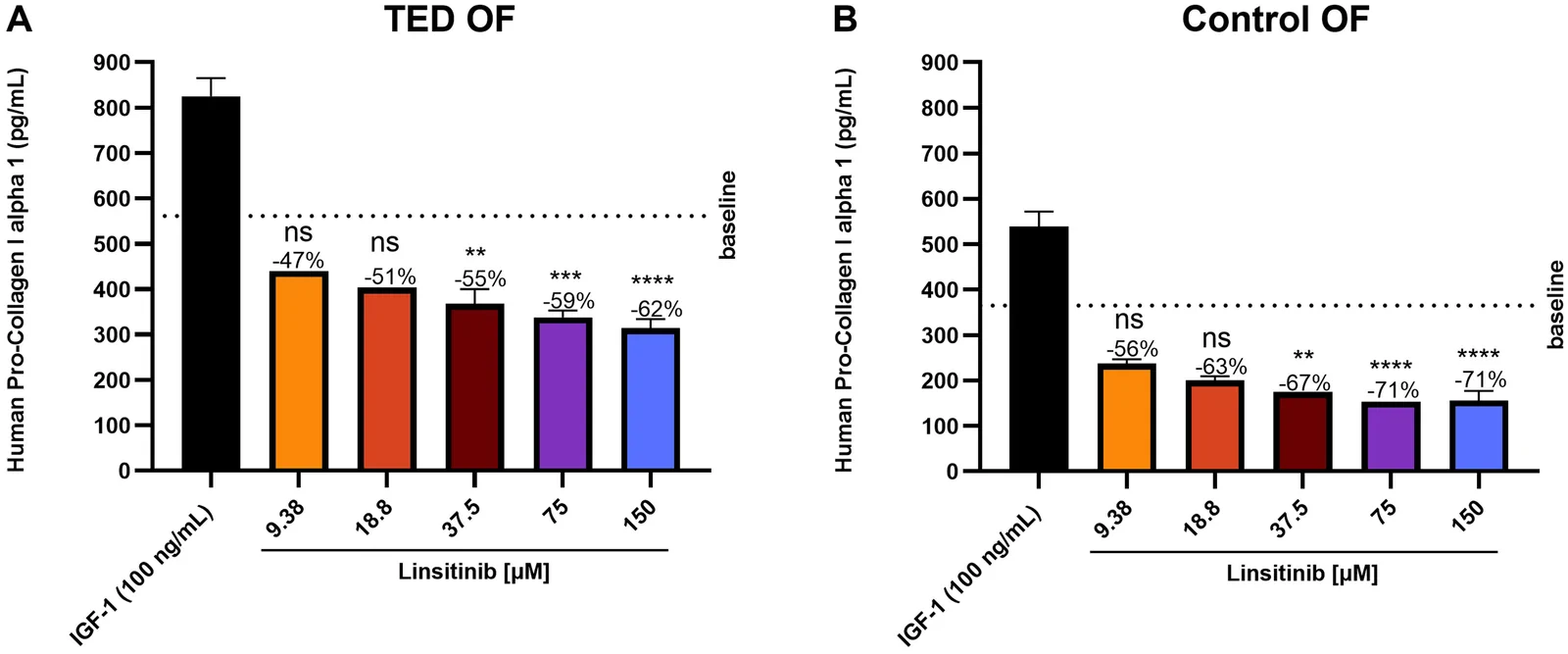
Linsitinib restores IGF-1-induced pro-collagen I release across TED and control orbital fibroblasts. Quantitative analysis of pro-collagen I concentration in the supernatant of primary human orbital fibroblasts (OF) from a TED patient **(A)** and a control **(B)**. After incubation with increasing concentrations of Linsitinib (9.38–150 μM) for 1 h, OF were stimulated with IGF-1 (100 ng/mL) for 48 h. **(A)** In TED OF, Linsitinib reduced pro-collagen release below baseline in a dose-dependent manner at 37.5 μM (−55%, P = 0.0080), 75 μM (−59%, P = 0.0003), and 150 μM (−62%, P <0.0001). **(B)** In control OF, reductions below baseline were achieved at 37.5 μM (−67%, P = 0.0079), 75 μM (−71%, P <0.0001), and 150 μM (−71%, P <0.0001). Data are expressed as absolute concentrations and presented as mean ± SD, with n = 6 replicates per concentration. Percentage changes and statistical significance were calculated relative to the IGF-1 positive control using the Kruskal–Wallis test followed by Dunn’s multiple comparisons test. ns, P >0.05. **P≤0.01. ***P≤0.001. ****P≤0.0001.

In a control donor, Linsitinib inhibited pro-collagen release in a comparable manner to that observed in TED donors (**Figure 3B**). Linsitinib treatment effectively suppressed this response up to 71%. Marked reductions below baseline were achieved at 37.5 μM (P = 0.0079), 75 μM (P <0.0001), and 150 μM (P <0.0001). Concentrations of 9.38 μM and 18.8 μM reduced secretion by 56% and 63%, respectively.

In another TED donor, stimulation with the IGF-1 positive control induced a minor increase in pro-collagen secretion (**Supplementary Figure 2A**). Linsitinib inhibited this stimulation up to 69%. A clear dose-dependent trend was observed, with higher concentrations resulting in stronger inhibition. Linsitinib restored pro-collagen secretion to levels below baseline at all tested concentrations: 37.5 μM (P = 0.0198), 75 μM (P = 0.0002), and 150 μM (P <0.0001). Concentrations of 9.38 μM and 18.8 μM reduced secretion by 53% and 59%, respectively. When another control donor was tested, IGF-1 positive control induced a robust increase in pro-collagen (**Supplementary Figure 2B**). Linsitinib inhibited this stimulation up to 58%. Secretion was reduced to below baseline level at 37.5 μM (P = 0.0003), 75 μM (P = 0.0002), and 150 μM (P = 0.0030).

#### 3.2.3 Linsitinib induces donor-specific morphological effects

Morphologically, compared to the negative control, strong stimulatory effects were observed following IGF-1 (100 ng/mL) stimulation in both TED and control OF (**Figure 4**). In the negative control, OF showed a standard quiescent state, forming a confluent monolayer. IGF-1 induced a clear morphological transition toward an activated state, characterized by markedly elongated, spindle-shaped, and stretched OF. This transition was morphologically evident in both TED and control OF. Linsitinib incubation resulted in clear, dose-dependent morphological changes in TED OF. While low concentrations of Linsitinib induced no visible changes, higher concentrations led to marked apoptotic effects compared to the negative control. At the highest concentration (150 μM), Linsitinib induced morphological characteristics of apoptosis. The OF showed a marked contracted and rounded shape, displaying a dark cellular body. This contraction resulted in numerous gaps compared to the confluent monolayer of the negative control. In addition, the stimulatory effects of IGF-1 were visibly inhibited compared to the positive control. At 75 μM, cells appeared contracted compared to both negative control and elongated positive control.

**Figure 4:**
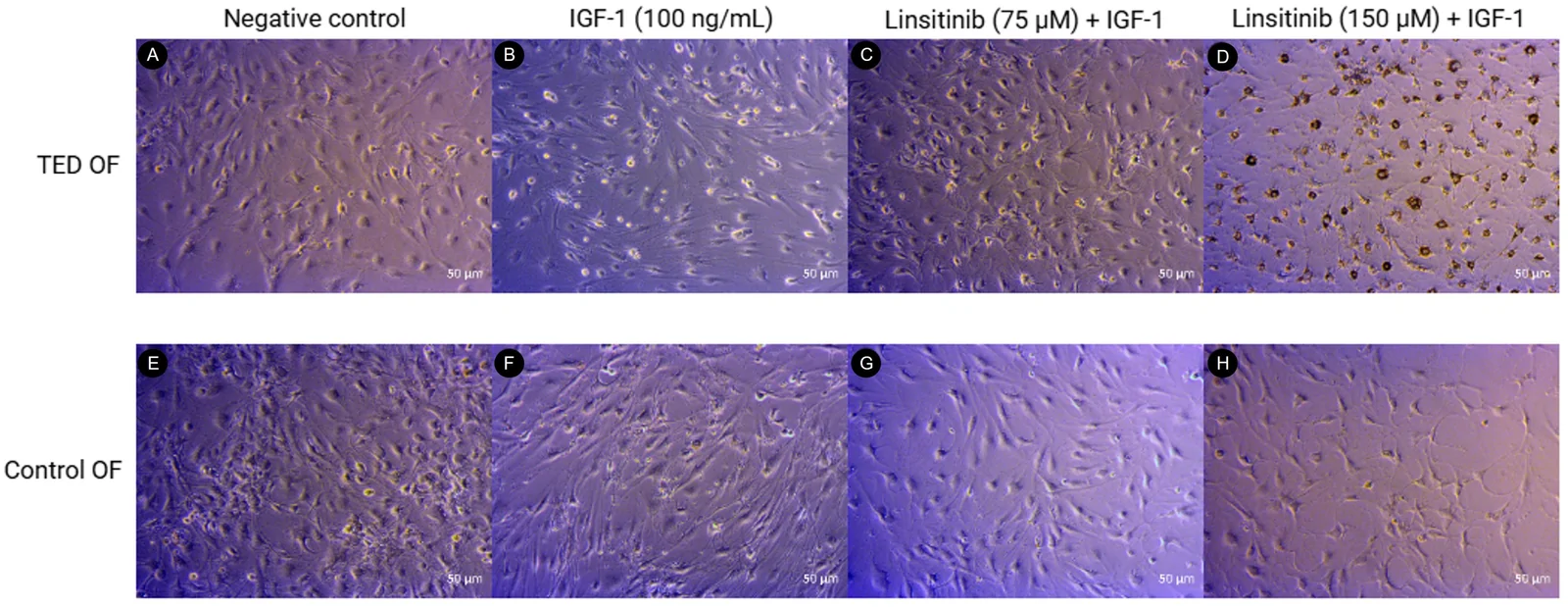
Linsitinib induces donor-specific morphological effects in primary human orbital fibroblasts. Representative phase-contrast microscopy images of primary human orbital fibroblasts (OF) from patients with thyroid eye disease (TED; **A–D**) and non-autoimmune controls **(E–H)**. After incubation with increasing concentrations of Linsitinib (9.38–150 μM) for 1 h, OF were stimulated with IGF-1 (100 ng/mL) for 48 h. **(A, E)** Untreated negative controls show a standard quiescent state, forming confluent monolayers. **(B, F)** Stimulation with IGF-1 (100 ng/mL) induces a transition toward an activated, elongated, and spindle-shaped morphology in both donor groups. **(C, G)** Treatment with 75 μM Linsitinib initiates cellular contraction, resulting in numerous gaps in both donor groups. **(D)** At 150 μM, Linsitinib induces morphological characteristics of apoptosis in TED OF. **(H)** Control OF show strong cellular contraction without apoptotic effects following treatment with 150 μM Linsitinib.

Compared to TED OF, Linsitinib treatment resulted in a different morphological profile in control OF. While high concentrations induced significant changes, no apoptotic effects were observed in contrast to TED donors. At the highest concentration of 150 μM, control OF showed strong cellular contraction. This was characterized by the formation of numerous gaps in the monolayer compared to the negative control. No significant morphological changes were observed at lower concentrations of Linsitinib.

### 3.3 Cell proliferation and apoptosis in primary orbital fibroblasts

#### 3.3.1 Linsitinib potently inhibits proliferation of orbital fibroblasts

Linsitinib significantly inhibited proliferation of primary OF from both TED and control donors in a clear dose-dependent manner, with higher concentrations resulting in stronger inhibition. In a TED donor (**Supplementary Figure 3A**), Linsitinib suppressed proliferation by up to 81% relative to control. Potent reductions were achieved at concentrations of 1.17 μM (P = 0.0487), 4.69 μM (P <0.0001), 9.38 μM (P = 0.0009), 18.8 μM (P = 0.0030), 37.5 μM (P <0.0001), 75 μM (P <0.0001), and 150 μM (P <0.0001). Concentrations of 0.293 μM and 0.586 μM reduced proliferation by 15% and 12%, respectively.

In another TED donor (**Supplementary Figure 3B**), inhibition reached a maximum of 70%. Linsitinib suppressed proliferation at concentrations of 2.34 μM (P = 0.0014), 4.69 μM (P <0.0001), 9.38 μM (P = 0.0044), 18.8 μM (P = 0.0003), 37.5 μM (P <0.0001), 75 μM (P <0.0001), and 150 μM (P <0.0001), while lower concentrations (0.293 μM, 0.586 μM, and 1.17 μM) led to minor reductions of 11%, 27%, and 26%, respectively.

Consistent with these findings in TED donors, Linsitinib inhibited proliferation of cells from a control donor (**Supplementary Figure 4**) in a comparable dose-dependent manner by up to 91%. Reductions were achieved at 1.17 μM (P = 0.0400), 4.69 μM (P = 0.0025), 9.38 μM (P <0.0001), 18.8 μM (P <0.0001), 37.5 μM (P <0.0001), 75 μM (P <0.0001), and 150 μM (P <0.0001). Concentrations of 0.293 μM and 0.586 μM reduced proliferation by 19% and 32%, respectively.

#### 3.3.2 Linsitinib slightly increases caspase-3/7 activity in orbital fibroblasts

Linsitinib slightly induced apoptosis in primary human OF from both TED and control donors. In a TED donor (**Supplementary Figure 5A**), Linsitinib increased caspase-3/7 activity by a maximum of 1.48-fold compared to control. Increases in this apoptosis marker were achieved at 0.293 μM (P = 0.0271), 0.586 μM (P = 0.0236), 1.17 μM (P = 0.0054), 2.34 μM (P = 0.0271), 75 μM (P = 0.0003), and 150 μM (P <0.0001), respectively.

In another TED donor (**Supplementary Figure 5B**), caspase-3/7 activity was enhanced by up to 1.47-fold relative to control, showing an effective increase at 18.8 μM (P = 0.0106), 37.5 μM (P = 0.0405), 75 μM (P <0.0001), and 150 μM (P <0.0001), respectively.

In a control donor (**Supplementary Figure 6**), Linsitinib increased caspase-3/7 activity by a maximum of 1.36-fold relative to control. Increases were achieved at 9.38 μM (P = 0.0243), 37.5 μM (P = 0.0146), 75 μM (P = 0.0001), and 150 μM (P <0.0001), respectively.

### 3.4 Equimolar comparison of Linsitinib and Teprotumumab

#### 3.4.1 Linsitinib induces dose-dependent morphological apoptosis

Morphological evaluation of IGF-1R-expressing cells treated with either Linsitinib or Teprotumumab (0.293 to 37.5 µM) demonstrated distinct differences in cellular response (**Figure 5**). Under control conditions, IGF-1R cells showed a standard quiescent state, forming a confluent monolayer. Linsitinib treatment induced clear, dose-dependent morphological changes. While lower concentrations showed no visible changes, moderate to high concentrations (9.38 to 37.5 µM) led to marked cell contraction, resulting in numerous gaps. These effects were most distinct at 37.5 μM, where cell contraction was significantly greater that that observed at lower doses. In contrast, Teprotumumab treatment showed no morphological changes across the entire tested concentration range. IGF-1R cells maintained a confluent monolayer comparable to the negative control.

**Figure 5:**
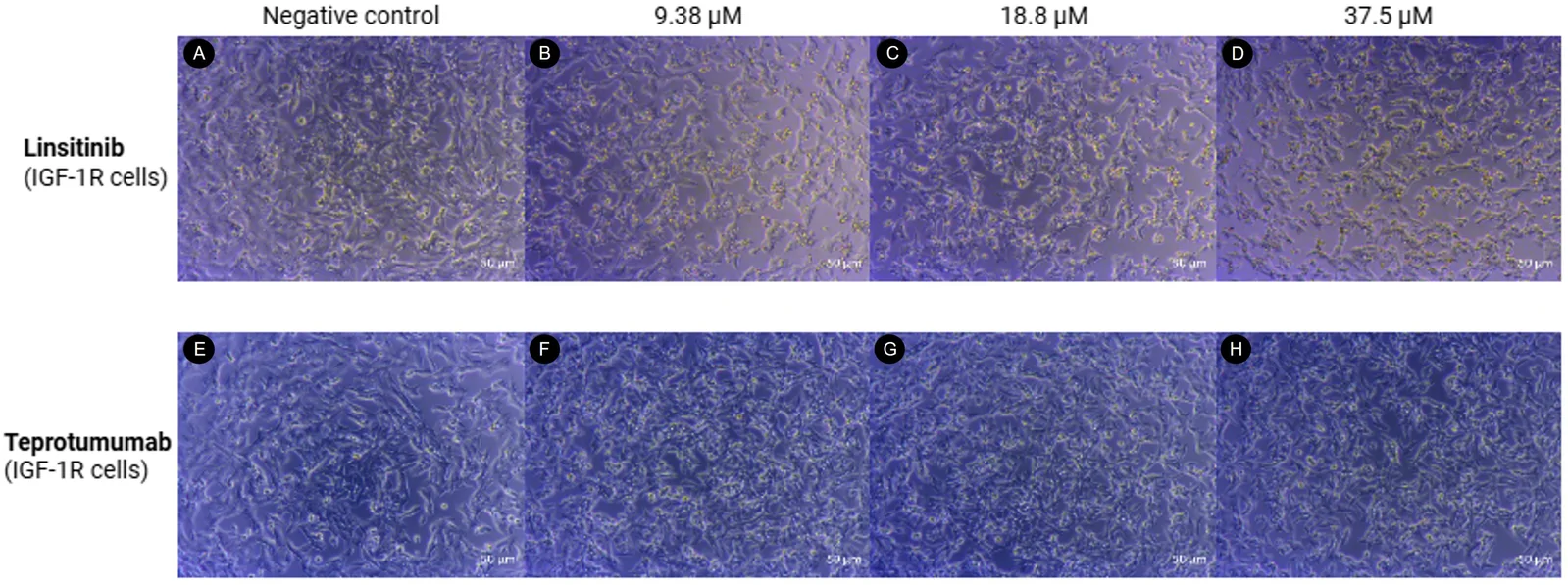
Distinct morphological effects of Linsitinib and Teprotumumab in IGF-1R-expressing cells. Representative phase-contrast microscopy images of IGF-1R-expressing cells following 24-h treatment with equimolar concentrations of either Linsitinib **(A–D)** or Teprotumumab **(E–H)**. **(A, E)** Untreated negative controls show a standard quiescent state, forming confluent monolayers. **(B–D)** Linsitinib induced dose-dependent morphological changes characterized by cellular contraction and formation of gaps in the monolayer, most pronounced at 37.5 μM. **(F–H)** Cells treated with Teprotumumab maintained a confluent monolayer and showed no morphological changes at any of the tested concentrations.

At additional very high concentrations of Linsitinib (75 μM and 150 μM), cells displayed clear morphological characteristics of apoptosis, characterized by a contracted, rounded shape and dark cellular bodies (**Supplementary Figure 7**). This contraction resulted in an extensive loss of the monolayer and a significant increase in gap formation compared with the negative control.

#### 3.4.2 Distinct apoptotic potential of Linsitinib compared to Teprotumumab

Linsitinib potently increased caspase-3/7 activity in IGF-1R-expressing cells, reaching a maximum 7.07-fold increase relative to control, indicating induction of apoptosis (**Figure 6A**). Increases were observed at 1.17 μM (P = 0.0014), 2.34 μM (P <0.0001), 4.69 μM (P <0.0001), 9.38 μM (P = 0.0287), 37.5 μM (P <0.0001), 75 μM (P <0.0001), and 150 μM (P <0.0001), respectively. In contrast, Teprotumumab showed no increase in caspase-3/7 activity at any of the tested concentration (**Figure 6B**).

**Figure 6:**
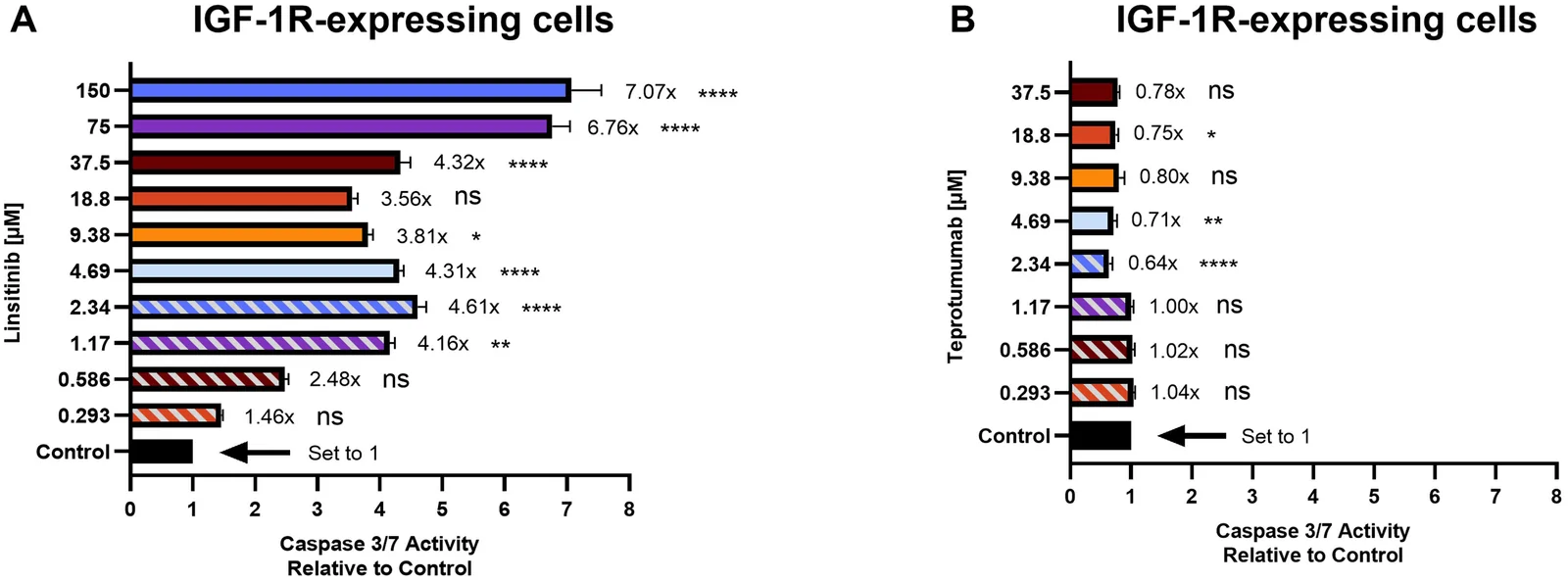
Linsitinib, but not Teprotumumab, induces robust activity of apoptosis marker caspase-3/7 in IGF-1R-expressing cells. Relative induction of apoptosis in IGF-1R-expressing cells measured by caspase-3/7 activity following 24-h treatment with equimolar concentrations of Linsitinib **(A)** or Teprotumumab **(B)**. For Linsitinib, two additional concentrations (up to 150 µM) were included. **(A)** Linsitinib increases caspase-3/7 activity at concentrations of 1.17 μM (4.16×, P = 0.0014), 2.34 μM (4.61×, P <0.0001), 4.69 μM (4.31×, P <0.0001), 9.38 μM (3.81×, P = 0.0287), 37.5 μM (4.32×, P <0.0001), 75 μM (6.76×, P <0.0001), and 150 μM (7.07×, P <0.0001), respectively. **(B)** In contrast, treatment with Teprotumumab showed no increase in caspase-3/7 activity at any concentration. Data are expressed relative to the untreated control (set to 1) and presented as mean ± SD, with n = 8 replicates per concentration. Relative changes and statistical significance were calculated relative to the control using the Kruskal–Wallis test followed by Dunn’s multiple comparisons test. ns, P >0.05. *P ≤0.05. **P ≤0.01. ****P ≤0.0001.

#### 3.4.3 Distinct inhibitory effects on cell proliferation by Linsitinib and Teprotumumab

Linsitinib potently inhibited the proliferation of IGF-1R-expressing cells in a dose-dependent manner, with higher concentrations resulting in stronger inhibition (**Figure 7A**). For the equimolar comparison (0.293–37.5 μM), Linsitinib inhibited proliferation by up to 65% relative to untreated control. Reductions were achieved at 1.17 μM (P = 0.0387), 2.34 μM (P = 0.0254), 4.69 μM (P = 0.0196), 9.38 μM (P = 0.0002), 18.8 μM (P <0.0001), and 37.5 μM (P <0.0001), respectively. Two additional very high concentrations further suppressed proliferation, reaching maximum inhibition at 75 μM and 150 μM, respectively (both P <0.0001).

**Figure 7:**
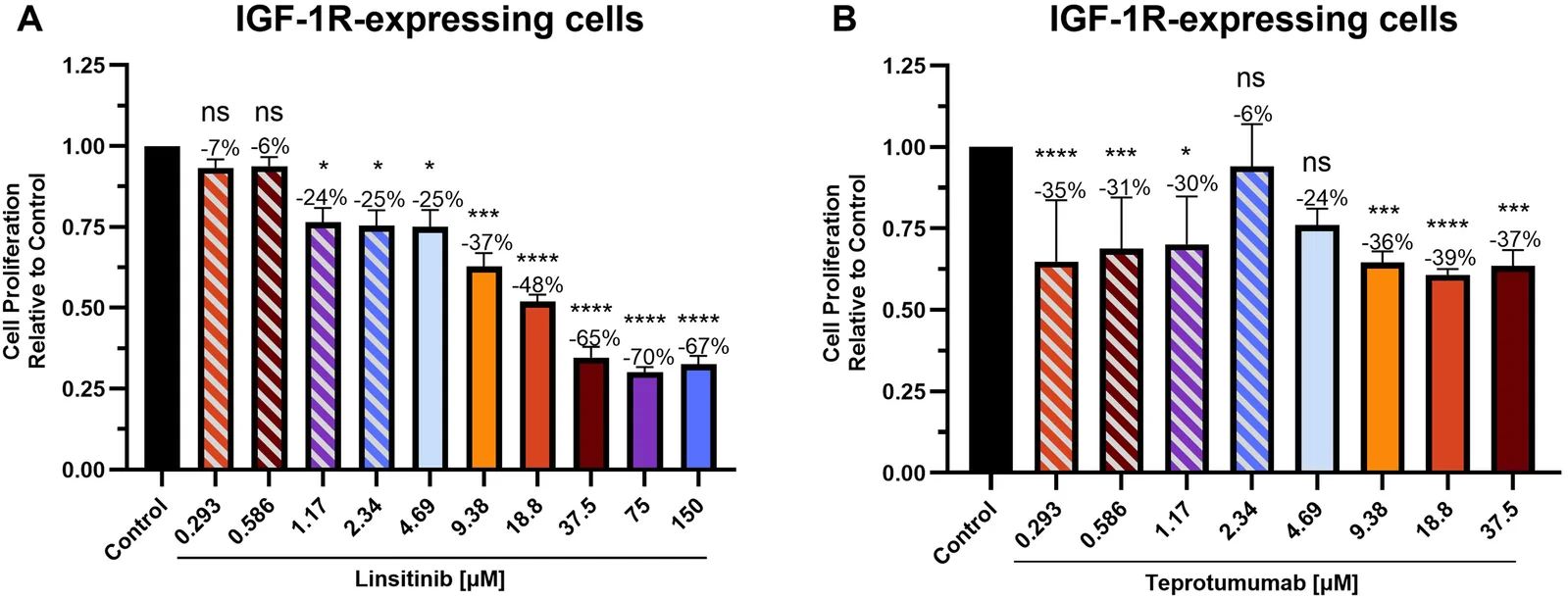
Distinct inhibitory effects on cell proliferation by Linsitinib and Teprotumumab in IGF-1R-expressing cells. Relative cell proliferation of IGF-1R-expressing cells following 24-h treatment with equimolar concentrations of Linsitinib **(A)** or Teprotumumab **(B)**. For Linsitinib, two additional concentrations (up to 150 µM) were included. **(A)** Linsitinib progressively inhibited cell proliferation in a dose-dependent manner: 1.17 μM (−24%, P = 0.0387), 2.34 μM (−25%, P = 0.0254), 4.69 μM (−25%, P = 0.0196), 9.38 μM (−37%, P = 0.0002), 18.8 μM (−48%, P <0.0001), and 37.5 μM (−65%, P <0.0001), respectively. Two additional higher concentrations further suppressed proliferation, −70% and −67% at 75 μM and 150 μM, respectively (both P <0.0001). **(B)** Cells treated with Teprotumumab showed inhibition at 0.293 μM (−35%, P <0.0001), 0.586 μM (−31%, P = 0.0006), and 1.17 μM (−30%, P = 0.0113), 9.38 μM (−36%, P = 0.0008), 18.8 μM (−39%, P <0.0001), and 37.5 μM (−37%, P = 0.0001), respectively. Data are expressed relative to the untreated control (set to 1.0) and presented as mean ± SD, with n = 8 replicates per concentration. Percentage changes and statistical significance were calculated relative to the control using the Kruskal–Wallis test followed by Dunn’s multiple comparisons test. ns, P >0.05. *P ≤0.05. ***P ≤0.001. ****P ≤0.0001.

Teprotumumab significantly inhibited proliferation at very low and high concentrations, with a maximum inhibition of 39% (**Figure 7B**). Marked inhibition was reached at the lowest concentrations of 0.293 μM (P <0.0001), 0.586 μM (P = 0.0006), 1.17 μM (P = 0.0113), and at high concentrations of 9.38 μM (P = 0.0008), 18.8 μM (P <0.0001), and 37.5 μM (P = 0.0001), respectively. In the direct (1:1) comparison (**Supplementary Figure 8**), Linsitinib suppressed proliferation more effectively than Teprotumumab at concentrations of 2.34 μM (P = 0.0019), 18.8 μM (P = 0.0002), and 37.5 μM (P = 0.0002), respectively. In contrast, Teprotumumab demonstrated higher potency at very low concentrations of 0.293 μM (P = 0.0392), 0.586 μM (P = 0.0101), and 1.17 μM (P = 0.0093), respectively.

## 4 Discussion

This innovative experimental work demonstrates the potent inhibitory effects of the IGF-1R blocker small-molecule Linsitinib on key pathological drivers of TED. The novel data were obtained using optimized physiological conditions, fresh human orbital tissue material, internationally acknowledged, standardized cell-biological methods, and universally established, frequently tested, specific assays. In primary human OF, Linsitinib effectively inhibited IGF-1-induced AKT (pS473) phosphorylation and markedly suppressed both IGF-1-induced HA and pro-collagen release. While HA release was significantly inhibited in both donor groups, TED OF showed greater sensitivity and apoptotic effects at high concentrations compared with control OF. The observed morphological effects were confirmed by a significant inhibition of cell proliferation and a slight increase in caspase-3/7 activity in both TED and control OF. In an equimolar comparison using IGF-1R-expressing cells, Linsitinib demonstrated a distinct profile from the mAb Teprotumumab, which has a higher molecular weight, marked by a fundamental difference in their impact on cell viability. Linsitinib markedly induced programmed cell death, whereas Teprotumumab failed to increase the activity of the apoptosis marker caspase-3/7 at any concentration. These apoptotic effects were most pronounced at high concentrations and were confirmed both morphologically and functionally by increased caspase-3/7 activity. Linsitinib also outperformed Teprotumumab across most tested concentrations.

Linsitinib (OSI-906) is a potent, oral, small-molecule inhibitor of both IGF-1R and IR, displaying a high degree of selectivity by fully blocking IGF-1-induced receptor autophosphorylation (26, 29). A phase III trial of Linsitinib in patients with locally advanced and/or metastatic adrenocortical carcinoma, as well as phase II studies (30, 31), reported evidence of antitumor activity in several solid tumors, with an acceptable tolerability profile (32–34). In the recent phase 2b/3, randomized, double-masked, placebo-controlled trial (NCT05276063) in patients with active, moderate to severe TED, Linsitinib met its primary endpoint of significant proptosis reduction (35). Mechanistically, the efficacy of Linsitinib derives from its ability to inhibit receptor autophosphorylation, thereby preventing the activation of the downstream signaling pathways PI3K/AKT and MAPK/ERK (26). Our present findings across both cellular models, including OF and IGF-1R-expressing cells, demonstrate the Linsitinib-induced blockade of these crucial pathways. Specifically, our findings show that Linsitinib suppresses IGF-1-induced AKT (pS473) phosphorylation in primary TED OF in a dose-dependent manner, restoring phosphorylation levels to baseline. This inhibition of the central pathway provides mechanistic support for the observed reduction of extracellular matrix secretion and the induction of apoptosis.

AKT, the central node of the PI3K/AKT pathway, is a highly conserved serine protein kinase that regulates downstream effectors responsible for controlling cell growth, cell cycle entry, and cell survival (11, 36–38). AKT consists of an N-terminal pleckstrin homology domain and a short C-terminal tail containing a regulatory hydrophobic motif (38). Full activation of AKT requires phosphorylation at both Thr308 and Ser473 in response to IGF-1 (39). The observed induction of apoptosis in both human TED OF and IGF-1R-expressing cells is strongly supported by the established antiapoptotic role of AKT. Specifically, AKT phosphorylates the Bcl-2-associated agonist of cell death (BAD), effectively blocking BAD-induced cell death (40). In line with our findings, an orally active inhibitor of IGF-1R induced apoptosis in the same IGF-1R cell line with a significant increase in caspase-3/7 activity (41). Furthermore, AKT mediates inhibition of Forkhead-box protein transcription factors, thereby preventing the expression of pro-apoptotic genes (10, 42). In addition, the serine/threonine kinase mammalian target of rapamycin (mTOR) is a key downstream effector protein of AKT (43). mTOR plays a central role in regulating critical cellular processes such as growth, proliferation, cytoskeletal organization, and protein synthesis (44). It exists in two distinct functional protein complexes (mTORC1 and mTORC2) (44). While mTORC1 is a critical regulator of cell growth, collagen synthesis, and collagen fibrillogenesis (45–48), mTORC2 is primarily involved in the activation of AKT (49).

The observed suppression of pro-collagen release in both TED and control OF is presumably attributable to this targeted inhibition of the AKT/mTOR axis by Linsitinib. Collagen is one of the most crucial components of the extracellular matrix, maintaining tissue and cellular shape, strength, and structural integrity (50). In the context of TED, OF secrete excessive amounts of collagen and other extracellular matrix components, which contribute to the pathological expansion of orbital fat and connective tissue (51–53). Interestingly, Linsitinib strongly inhibited the proliferation of primary OF from TED and control donors while only slightly increasing caspase-3/7 activity. This supports its potential role in TED by suppressing pathological tissue expansion without inducing widespread cell death.

The marked inhibition of cell proliferation observed with Linsitinib in IGF-1R-expressing cells can be attributed to the suppression of mTORC1, which regulates cell growth, and, more importantly, to the blockade of ERK1 and ERK2 (ERK1/2). ERK1/2 are the terminal components of the MAPK/ERK pathway (43). ERK1/2 are key regulators of cell proliferation, while ERK2 has a dominant role in regulating proliferation due to its markedly higher expression level compared with that of ERK1 (54). Consistent with our results, comparably potent inhibition of proliferation in the same IGF-1R-expressing cell line was reported using the small molecule IGF-1R blockers AEW-541 and Linsitinib, respectively (55, 56). Furthermore, Linsitinib effectively prevented phosphorylation of the critical residue Tyr1135 within the IGF-1R β-subunit and ERK, leading to the subsequent downregulation of cell proliferation in TED OF (57).

Our equimolar comparison demonstrated distinct effects of Linsitinib and the mAb Teprotumumab with regard to their inhibition of cell proliferation and induction of apoptosis. As an ATP-competitive kinase inhibitor, Linsitinib targets the intracellular domain of the receptor (β-subunit), whereas Teprotumumab is restricted to the extracellular domain or α-subunit due to its markedly higher molecular weight. Furthermore, Linsitinib potently inhibits both IGF-1R and IR, preventing the activation of the PI3K/AKT and MAPK/ERK pathways (26). We therefore speculate that this dual inhibition may provide a more robust suppression of downstream signaling than that provided by Teprotumumab competing with the ligand IGF-1 for binding to the cysteine-rich domain of the IGF-1R α-subunit. In line with this, Linsitinib markedly decreased phosphorylation of IGF-1R by >90% and of IR by >50% in human tumor cell lines, whereas an IGF-1R blocking mAb was similarly effective at IGF-1R but only moderately effective at IR in one of nine tested tumor cell lines (58). Interestingly, treatment with the IGF-1R-blocking mAb resulted in a substantial increase in phosphorylation of IR (58). This supports Linsitinib’s potentially stronger effects in inhibiting proliferation and inducing apoptosis as the dual blockade prevents signaling through the IR observed with mAb treatment. In addition, although IGF-1R blocking mAbs, e.g., Teprotumumab, have a much higher target affinity (fmol/L to pmol/L) than small molecules (nmol/L to μmol/L) (23), Teprotumumab is a competitive inhibitor at the extracellular domain of the IGF-1R. The IGF-1R cell line NCI-H295R is known to secrete large amounts of IGF-2, which increase over time in culture (59). These large amounts of IGF-2 enhance competition for extracellular receptor binding, potentially hindering Teprotumumab from binding to the receptor. In contrast, the intracellular site of action of Linsitinib allows effective prevention of kinase activity regardless of the high extracellular ligand concentration.

Moreover, besides its role in cell proliferation, the activation of ERK is a driver of HA synthase (HAS) activity (60, 61), leading to marked release of the hydrophilic hyaluronan macromolecule by activated OF, inducing local edema and soft tissue swelling (18, 53, 62). The present findings demonstrate that Linsitinib markedly suppresses IGF-1-induced HA release in TED OF, in line with other reports (57, 63).

The authors acknowledge the following limitations: (A) For the HA and pro-collagen assays, OF were stimulated with IGF-1 only, disregarding the established TSH-R/IGF-1R crosstalk. This synergistic interaction relies on TSH-R activation to enhance HA secretion (15, 18). Therefore, future experiments should evaluate this crosstalk by stimulating cells with the TSH-R agonist mAb M22 and/or the combination of M22 and IGF-1. Experimentally, Linsitinib has demonstrated strong inhibition of TSH-R/IGF-1R crosstalk in TED OF, evidenced by suppression of M22-stimulated HA secretion (15). (B) In addition, thyroid-stimulating immunoglobulins (TSI) are a specific and highly prevalent biomarker in TED (64). Hence, OF should also be stimulated with TSI-positive TED sera. (C) While our equimolar comparison of Linsitinib and Teprotumumab was performed in an IGF-1R-expressing cell line, these results should be confirmed in primary human OF. (D) Simultaneous targeting of both extracellular and intracellular domains of the IGF-1R seems worth testing. (E) Interindividual variability observed across tested donors *in vitro* may be attributed to differences in disease activity, severity, and previous immunomodulating treatments.

In summary, Linsitinib treatment suppressed IGF-1-induced AKT phosphorylation to baseline levels and effectively restored levels of HA and pro-collagen release in primary human OF. In the equimolar comparison, Linsitinib significantly outperformed Teprotumumab in inhibiting cell proliferation and inducing apoptosis. These findings highlight Linsitinib as a potent, dual-targeted therapy for TED.

## Data Availability

The original contributions presented in the study are included in the article/**Supplementary Material**. Further inquiries can be directed to the corresponding author.

## Supplementary material

Supplementary Figure 1 Linsitinib restores IGF-1-induced hyaluronan secretion with enhanced efficacy in TED orbital fibroblasts. Quantitative analysis of hyaluronic acid (HA) concentration in the supernatant of primary human orbital fibroblasts (OF) from TED patient **(A)** and healthy control **(B)**. After incubation with rising concentrations of Linsitinib (9.38-150 μM) for 1h, OF were stimulated with IGF-1 (100 ng/mL) for 48 h. **(A)** In TED OF, Linsitinib suppressed HA secretion to baseline level at concentrations of 9.38 μM (-58%, P = 0.0027), 18.8 μM (-56%, P = 0.0330), 37.5 μM (-56%, P = 0.198), and 150 μM (-61%, P = 0.0022), respectively. **(B)** In control OF, Linsitinib reduced HA secretion below baseline only at the highest concentration of 150 μM (-37%, P = 0.0197). Data are expressed as absolute concentrations and presented as mean + SD with n=6 replicates per concentration. Percentage changes and statistical significance were calculated relative to the IGF-1 positive control using Kruskal-Wallis test followed by Dunn’s multiple comparisons test. ns means P>0.05. *P ≤ 0.05. **P ≤ 0.01.

Supplementary Figure 2 Linsitinib suppresses IGF-1-induced pro-collagen I release across TED and control orbital fibroblasts. Quantitative analysis of pro-collagen I concentration in the supernatant of primary human orbital fibroblasts (OF) from TED patient **(A)** and healthy control **(B)**. After incubation with rising concentrations of Linsitinib (9.38-150 μM) for 1h, OF were stimulated with IGF-1 (100 ng/mL) for 48 h. **(A)** In TED OF, Linsitinib restored pro-collagen secretion to levels below baseline at the following concentrations: 37.5 μM (-62%, P = 0.0198), 75 μM (-67%, P = 0.0002), and 150 μM (-69%, P<0.0001), respectively. **(A)** In control OF, Linsitinib reduced secretion to below baseline level at 37.5 μM (-58%, P = 0.0003), 75 μM (-58%, P = 0.0002), and 150 μM (-56%, P = 0.0030), respectively. Data are expressed as absolute concentrations and presented as mean + SD with n=6 replicates per concentration. Percentage changes and statistical significance were calculated relative to the IGF-1 positive control using Kruskal-Wallis test followed by Dunn’s multiple comparisons test. ns P>0.05. *P ≤ 0.05. **P ≤ 0.01. ***P ≤ 0.001. ****P ≤ 0.0001.

Supplementary Figure 3 Linsitinib inhibits proliferation in TED orbital fibroblasts. Relative cell proliferation of primary human orbital fibroblasts (OF) from TED patients **(A, B)** following 24 h treatment with rising concentrations of Linsitinib (0.293-150 μM). **(A)** In a TED donor, Linsitinib suppressed proliferation effectively at 1.17 μM (P = 0.0487), 4.69 μM (P<0.0001), 9.38 μM (P = 0.0009), 18.8 μM (P = 0.0030), 37.5 μM (P<0.0001), 75 μM (P<0.0001), and 150 μM (P<0.0001), respectively. **(B)** In another TED donor, inhibition of proliferation was effective at concentrations of 2.34 μM (P = 0.0014), 4.69 μM (P<0.0001), 9.38 μM (P = 0.0044), 18.8 μM (P = 0.0003), 37.5 μM (P<0.0001), 75 μM (P<0.0001), and 150 μM (P<0.0001), respectively. Data are expressed relative to the untreated control (set to 1.0) and presented as mean + SD with n=8 replicates per concentration. Percentage changes and statistical significance were calculated relative to the control using Kruskal-Wallis test followed by Dunn’s multiple comparisons test. ns P>0.05. *P ≤ 0.05. **P ≤ 0.01. ***P ≤ 0.001. ****P ≤ 0.0001.

Supplementary Figure 4 Linsitinib inhibits proliferation in control orbital fibroblasts. Relative cell proliferation of primary human orbital fibroblasts (OF) from a control patient following 24 h treatment with rising concentrations of Linsitinib (0.293-150 μM). Linsitinib suppressed proliferation effectively at 1.17 μM (P = 0.0400), 4.69 μM (P = 0.0025), 9.38 μM (P<0.0001), 18.8 μM (P<0.0001), 37.5 μM (P<0.0001), 75 μM (P<0.0001), and 150 μM (P<0.0001), respectively. Data are expressed relative to the untreated control (set to 1.0) and presented as mean + SD with n=8 replicates per concentration. Percentage changes and statistical significance were calculated relative to the untreated control using Kruskal-Wallis test followed by Dunn’s multiple comparisons test. ns P>0.05. *P ≤ 0.05. **P ≤ 0.01. ****P ≤ 0.0001.

Supplementary Figure 5 Linsitinib slightly increases caspase-3/7 activity in TED orbital fibroblasts. Relative induction of apoptosis in primary human orbital fibroblasts (OF) from TED patients **(A, B)** measured by caspase-3/7 activity following 24 h treatment with rising concentrations of Linsitinib (0.293-150 μM). (**A**) In a TED donor, Linsitinib slightly increased caspase-3/7 activity effectively at concentrations of 0.293 μM (1.09x, P = 0.0271), 0.586 μM (1.10x, P = 0.0236), 1.17 μM (1.11x, P = 0.0054), 2.34 μM (1.10x, P = 0.0271), 75 μM (1.15x, P = 0.0003), and 150 μM (1.48x, P<0.0001), respectively. **(B)** In another TED donor, Linsitinib slightly increased caspase-3/7 activity effectively at 18.8 μM (1.12x, P = 0.0106), 37.5 μM (1.10x, P = 0.0405), 75 μM (1.31x, P<0.0001), and 150 μM (1.47x, P<0.0001), respectively. Data are expressed relative to the untreated control (set to 1.0) and presented as mean + SD with n=8 replicates per concentration. Relative changes and statistical significance were calculated relative to the control using Kruskal-Wallis test followed by Dunn’s multiple comparisons test. ns P>0.05. *P ≤ 0.05. **P ≤ 0.01. ***P ≤ 0.001. ****P ≤ 0.0001.

Supplementary Figure 6 Linsitinib slightly increases caspase-3/7 activity in control orbital fibroblasts. Relative induction of apoptosis in primary human orbital fibroblasts (OF) from a control patient measured by caspase-3/7 activity following 24 h treatment with rising concentrations of Linsitinib (0.293-150 μM). Linsitinib slightly increased caspase-3/7 activity effectively at concentrations of 9.38 μM (1.11x, P = 0.0243), 37.5 μM (1.12x, P = 0.0146), 75 μM (1.20x, P = 0.0001), and 150 μM (1.36x, P<0.0001), respectively. Data are expressed relative to the untreated control (set to 1.0) and presented as mean + SD with n=8 replicates per concentration. Relative changes and statistical significance were calculated relative to the control using Kruskal-Wallis test followed by Dunn’s multiple comparisons test. ns P>0.05. *P ≤ 0.05. ***P ≤ 0.001. ****P ≤ 0.0001.

Supplementary Figure 7 Linsitinib induces morphologically confirmed apoptosis in IGF-1R-expressing cells. Representative phase-contrast microscopy images of IGF-1R-expressing cells following 24 h treatment with additional doses of Linsitinib. **(A)** Untreated negative control showed a standard quiescent state, forming confluent monolayer. **(B, C)** At very high concentrations of Linsitinib (75 μM and 150 μM), cells displayed clear morphological characteristics of apoptosis, characterized by a contracted, rounded shape, and dark cellular bodies. This contraction resulted in an extensive loss of monolayer and significant formation of gaps compared to the negative control.

Supplementary Figure 8 Equimolar (1:1) comparison of Linsitinib and Teprotumumab on cell proliferation in IGF-1R-expressing cells. Relative cell proliferation of IGF-1R-expressing cells following 24 h treatment with equimolar concentrations of Linsitinib and Teprotumumab. Linsitinib suppressed proliferation more effectively than Teprotumumab at concentrations 2.34 μM (P = 0.0019), 18.8 μM (P = 0.0002), and 37.5 μM (P = 0.0002), respectively. In contrast, compared to Linsitinib, Teprotumumab demonstrated higher potency at very low concentrations: 0.293 μM (P = 0.0392), 0.586 μM (P = 0.0101), and 1.17 μM (P = 0.0093), respectively. Data are expressed relative to the untreated control (set to 1.0) and presented as mean + SD with n=8 replicates per concentration. Statistical significance between Linsitinib and Teprotumumab at each concentration was calculated using the two-tailed Mann-Whitney test. ns P>0.05. *P ≤ 0.05. ** P ≤ 0.01. ***P ≤ 0.001.
